# The expression of selenium-binding protein 1 is decreased in uterine leiomyoma

**DOI:** 10.1186/1746-1596-5-80

**Published:** 2010-12-09

**Authors:** Peng Zhang, Cunxian Zhang, Xudong Wang, Fang Liu, C James Sung, M Ruhul Quddus, W Dwayne Lawrence

**Affiliations:** 1 Department of Pathology and Laboratory Medicine, Women & Infants Hospital of Rhode Island, Warren Alpert Medical School of Brown University, 101 Dudley Street, Providence, Rhode Island 02905, USA

## Abstract

**Background:**

Selenium has been shown to inhibit cancer development and growth through the mediation of selenium-binding proteins. Decreased expression of selenium-binding protein 1 has been reported in cancers of the prostate, stomach, colon, and lungs. No information, however, is available concerning the roles of selenium-binding protein 1 in uterine leiomyoma.

**Methods:**

Using Western Blot analysis and immunohistochemistry, we examined the expression of selenium-binding protein 1 in uterine leiomyoma and normal myometrium in 20 patients who had undergone hysterectomy for uterine leiomyoma.

**Results and Discussion:**

The patient age ranged from 34 to 58 years with a mean of 44.3 years. Proliferative endometrium was seen in 8 patients, secretory endometrium in 7 patients, and atrophic endometrium in 5 patients. Two patients showed solitary leiomyoma, and eighteen patients revealed 2 to 5 tumors. Tumor size ranged from 1 to 15.5 cm with a mean of 4.3 cm. Both Western Blot analysis and immunohistochemistry showed a significant lower level of selenium-binding protein 1 in leiomyoma than in normal myometrium. Larger tumors had a tendency to show a lower level of selenium-binding protein 1 than smaller ones, but the difference did not reach a statistical significance. The expression of selenium-binding protein 1 was the same among patients with proliferative, secretory, and atrophic endometrium in either leiomyoma or normal myometrium. Also, we did not find a difference of selenium-binding protein 1 level between patients younger than 45 years and older patients in either leiomyoma or normal myometrium.

**Conclusions:**

Decreased expression of selenium-binding protein 1 in uterine leiomyoma may indicate a role of the protein in tumorigenesis. Our findings may provide a basis for future studies concerning the molecular mechanisms of selenium-binding protein 1 in tumorigenesis as well as the possible use of selenium in prevention and treatment of uterine leiomyoma.

## Introduction

Uterine leiomyoma, the most common neoplasm of the female genital tract, probably occurs in the majority of women by age 50 and is responsible for significant morbidity in patients [1-3]. Symptoms include pelvic pressure, pelvic pain, abnormal uterine bleeding, infertility, and miscarriage [4,5]. Uterine leiomyoma represents a major indication for hysterectomy among women in the United States, accounting for one-third of about 600,000 hysterectomy procedures performed annually [6,7]. Not only is hysterectomy associated with morbidity and mortality, but it also has a huge economic impact on healthcare systems [1].

The scientific literature contains a large body of information concerning the epidemiology, hormonal influence, genetics, and molecular alterations in uterine leiomyoma. Risk factors include early menarche, nulliparity, obesity, African-American ethnicity, and temoxifen use [8-13]. Many of these factors are associated with increased levels of estrogen and progesterone. Estrogen and progesterone act through the mediation of estrogen receptor and progesterone receptor, respectively. The majority of literature revealed higher concentrations of estrogen and progesterone receptors in leiomyoma than in normal myometrium [3]. Leiomyoma of the uterus also overexpresses various growth factors including transforming growth factor, fibroblastic growth factor, epidermal growth factor receptor, and platelet-derived growth factor [3].

Inherent abnormality of myometrium in patients has also been implicated in the development of leiomyoma since the myometrium in the uterus harboring leiomyoma shows a significantly higher level of estrogen receptor than that without tumor [14]. Leiomyoma of the uterus has been shown to be monoclonal by studies using X-linked glucose 6-phosphate dehydrogenase isozymes [15], X-linked androgen receptor [16,17], and X-linked phosphoglycerokinase [18]. Cytogenetic studies have identified several chromosomal alterations, including t(12;14), del(7q), 6p21, and trisomy 12 (3). However, it is unclear whether the genetic alterations occur before the genesis of leiomyoma or they are secondary events.

Despite numerous studies concerning the molecular and genetic changes in uterine leiomyoma, the mechanisms of development remain unknown. Further work is needed to elucidate the pathogenesis that would lead to the discovery of effective prevention and treatment of the tumor.

Selenium, an essential trace element, has been shown to have an anti-cancer effect. Many reports have described a relationship between insufficient selenium intake and increased risk of cancer [19-21]. The anti-cancer action of selenium is thought to be mediated by selenium-binding protein 1 (SELENBP1), a 56 kDa intracellular protein, that binds covalently to selenium. The gene of SELENBP1 is located at chromosome 1q21-22 [22]. The expression of SELENBP1 has been shown to be decreased in several tumors including cancers of the prostate, lungs, colon, and ovary [23-26]. However, little information exists concerning the role of SELENBP1 in tumorigenesis of uterine leiomyoma. In this study, we examined the expression of SELENBP1 in uterine leiomyoma and normal myometrium.

## Materials and methods

The study consisted of 20 consecutive specimens of hysterectomy performed for leiomyoma at our institution in July 2004. We recorded the number and size of leiomyoma as well as the endometrial pattern in each patient. Using a monoclonal antibody against human SELENBP1 (Medical Biological Laboratory International Corporation, Watertown, MA), we evaluated the expression of SELENBP1 by Western Blot and immunohistochemistry.

For Western Blot, 100 mg sample was taken from each leiomyoma of an unfixed uterine specimen. We selected areas of leiomyoma without degenerative changes. Also sampled was 100 mg of tissue from normal myometrium in the same uterus. The sample was immediately placed in 1 ml radioimmunoprecipitation assay buffer containing 50 mM Tris-HCl (pH7.4), 150 mM NaCl, 1% Triton X-100, 1% sodium deoxycholate, 0.1% SDS, 1 mM PMSF, 1 mM EDTA, 5 ug/ml aprotinin, 5 ug/ml leupeptin, 1 mM Na_3_VO_4_, and 5 mM NaF. After being cut into smaller pieces with scissors, the sample was homogenized on ice with a motor-driven tissue Tearor for 5 times, each for 10 seconds. The homogenate was placed on an orbital shaker at 4°C for 30 minutes and then centrifuged at 4°C with 14,000 × g for 15 minutes. The supernatant was collected in a fresh tube and stored at -80°C for later use.

Protein concentration was determined by a Bradford protein assay (Bio-Rad, Hercules, CA), using bovine serum albumin as standard. We heated each sample at 95°C for 5 minutes after mixing it with Laemmli sample buffer containing 62.5 mM Tris-HCl (pH6.8), 20% glycerol, 2% SDS, 0.01% bromophenol blue, and 5% β-mercaptoethanol. Equal amount of protein (25 μg) from every sample was separated on 12% sodium dodecyl sulfate polyacrylamide (Tris/glycine) gel and transferred to polyvinylidene difluoride membrane. After being stained with Ponceau S, the membrane was blocked in phosphate-buffered saline containing 5% nonfat dry milk for 30 minutes, and incubated with antibodies against SELENBP1 at a dilution of 1:400 and β-actin (Santa Cruz Biotechnology, Santa Cruz, CA) at a dilution of 1:200 for 1 hour. After three washes in phosphate-buffered saline with 0.1% Tween 20, the membranes were incubated with horse radish peroxidase-conjugated anti-mouse immunoglobulin G (Medical Biological Laboratory International Corporation, Watertown, MA) at a dilution of 1:10,000 for 1 hour, followed by enhanced chemiluminescence detection (Thermo Scientific, Waltham, MA) and exposure to an X-ray film. Relative abundance of protein was determined by quantitative densitometry using the National Institutes of Health image program (available at http://rsb.info.nih.gov/nih-image/). Molecular weights of proteins were determined by extrapolation from the relative mobility of known molecular weights. All Western Blot densitometry data on SELENBP1 were normalized to β-actin (a house keeping protein). The relative level of SELENBP1 was then normalized by the mean level of SELENBP1 in normal myometrium.

To verify the result of Western Blot analysis, we performed immunohistochemistry in archival tissue. Five micron sections were taken from paraffin blocks of leiomyoma and normal myometrium of the same uterine specimens used for Western Blot analysis. As in Western Blot, we selected sections of leiomyoma that did not show degenerative changes such as infarction or hyalinization. The sections were routinely deparaffinized and hydrated through a gradient of ethanol. Antigen retrieval was achieved by incubating the sections with citrate buffer (pH 6.1) at 95°C. Sections were immersed in 3% H_2_O_2 _at room temperature for 10 minutes to block any endogenous peroxidase activity. They were incubated with SELENBP1 antibody at a dilution of 1:200 at room temperature for 35 minutes. The sections were then incubated with a secondary antibody previously conjugated to horseradish peroxidase-labeled polymer at room temperature for 35 minutes. After the sections were incubated with diaminobenzidine and H_2_O_2 _at room temperature for 8 minutes, they were counterstained with hematoxylin and cover-slipped.

In each run of immunohistochemistry, we included several controls: (1) a negative reagent control (Medical Biological Laboratory International Corporation, Watertown, MA), used to substitute the primary antibody, (2) a positive tissue control with a section of normal fallopian tube known to be positive for SELENBP1, and (3) a negative tissue control with a section of high-grade ovarian serous carcinoma known to be negative for SELENBP1. The specificity of immunostaining was confirmed by a positive stain in the mucosal epithelial cells of fallopian tube but a negative stain in high-grade ovarian serous carcinoma and on the sections that were stained with a negative reagent control.

The immunostains were scored using a 4-point scale (0-3+) system, based on the number of positive cells and the intensity of staining; no staining was recorded as "0", weak staining in fewer than one-third of cells as "1", moderate staining in one-third to two-thirds of cells as "2", and strong staining in more than two-thirds of cells as "3".

The immunostaining scores of SELENBP1 in leiomyomas were correlated with vascular count on hematoxylin and eosin stained sections of leiomyomas originating from the same paraffin blocks used for SELENBP1 immunostain. Vascular count was defined as the number of blood vessels in 5 consecutive low power microscopic fields because each section of leiomyoma contained a minimum of 5 low power fields for evaluation. To assess proliferation index relative to SELENBP1 expression, the proliferation index was determined in sections of leiomyomas using MIB-1 antibody to the Ki67 antigen (Dako, Carpinteria, CA). When evaluating the results of Ki67 immunostaining, we chose the tumor area with the highest density of positive nuclear staining. A minimum of 200 cells on each section of leiomyoma were analyzed. The proliferation index, represented by the percentage of positive nuclei, was calculated by dividing the number of positive stained cells by the total number of cells in the areas examined. Pearson correlation coefficient analysis was used to evaluate the relationship between vascular count and SELENBP1 immunostaining score as well as proliferation index and SELENBP1 immunostaining score. When the p value was smaller than 0.05, the relationship was considered significant.

Wilcoxon Matched-Pair Signed-Ranks test was used to compare the expression of SELENBP1 between leiomyoma and normal myometrium. For cases showing more than one leiomyoma, the average SELENBP1 level of multiple tumors in the same patient was used to compare with the SELENBP1 level of normal myometrium. In order to evaluate whether patient's age was related to the level of SELENBP1, we divided patients into two arbitrary age groups: <45 years and ≥45 years. The abundance of SELENBP1 in the two age groups was compared with Mann-Whitney test. To determine whether the level of SELENBP1 was related to the size of leiomyoma, tumors were divided into four arbitrary size groups: ≤2 cm, 2.1-5 cm, 5.1-8 cm, and ≥8 cm. The levels of SELENBP1 in the four size groups were compared by Analysis of Variance. Analysis of Variance was also used to compare the abundance of SELENBP1 among patients that showed proliferative, secretory, and atrophic endometrium. The difference was considered significant when the p value was smaller than 0.05.

## Results

Patient characteristics are shown in Table 1. The patient age ranged from 34 to 58 years, with a mean of 44.3 years. There were 8 patients with proliferative endometrium, 7 with secretory endometrium, and 5 with atrophic endometrium. Two patients displayed solitary leiomyoma, and eighteen patients showed 2 to 5 tumors. The size of leiomyoma varied from 1 to 15.5 cm, with a mean of 4.3 cm.

**Table 1:** Characteristics of patients

Case number	Patient's age (years)	Endometrial pattern	Number of leiomyoma	Tumor size (cm)
1	43	P	1	6.5

2	50	A	4	1, 2, 3.5, 6

3	46	P	4	1.5, 2.5, 4, 7.5

4	49	S	3	2, 8, 12

5	34	P	5	3, 3, 4.5, 5, 15.5

6	38	S	3	1.5, 4, 6.8

7	40	S	1	5.5

8	39	P	5	1, 1.5, 2, 2, 11.8

9	36	S	4	2, 3.5, 4, 11.5

10	41	P	5	1.5, 2, 3.5, 4, 10.5

11	57	A	3	2, 5.5, 9.5

12	37	P	2	1.5, 7.5

13	45	P	5	1, 2.5, 2.5, 4, 9

14	55	A	5	2, 2, 3.5, 4, 5

15	53	A	4	1, 2, 3, 6.5

16	44	S	4	2, 2.5, 4, 5

17	58	A	4	2.5, 4, 4.5, 9

18	38	S	4	3.5, 3.5, 4, 6.3

19	42	S	4	2, 2.5, 3, 4.5

20	40	P	3	1, 3, 5.5

Mean	44.3	N/A	3.6	4.3

Abbreviations: P, proliferative; S, secretory; A, atrophic; N/A, not applicable

**Figure 1:**
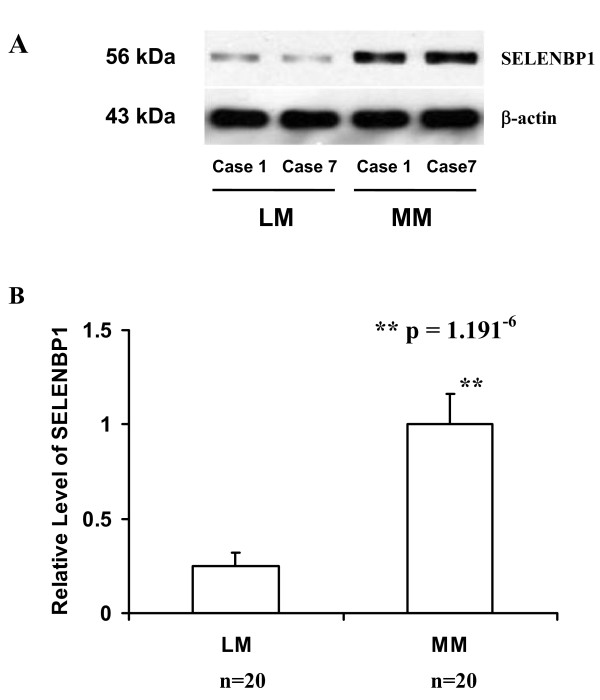
**Western Blot analysis of SELENBP1 expression in uterine leiomyoma and normal myometrium**. (A) The bands of SELENBP1 in two examples displayed about 4-fold lower density in leiomyoma than in normal myometrium. (B) After normalizing for β-actin and then for the mean of SELENBP1 in normal myometrium, data derived from densitometry of Western Blot experiments were used to represent relative levels of SELENBP1. Wilcoxon Signed-Ranked test for Matched Pairs showed a significant decrease of SELENBP1 level in leiomyoma compared to that in normal myometrium. The vertical bars in B represent means and standard deviations. Abbreviations: LM, leiomyoma; MM, normal myometrium; SELENBP1, selenium-binding protein1 1; n, number of cases.

**Figure 2:**
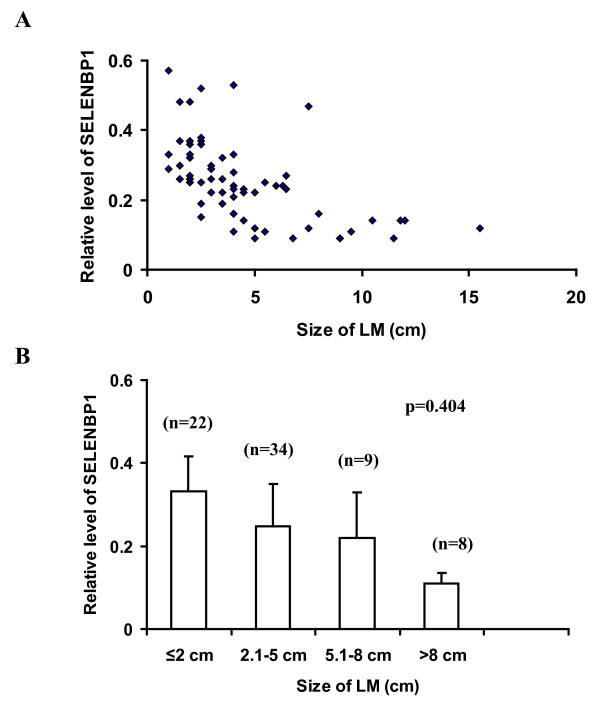
**Relationship between size of leiomyoma and level of SELENBP1**. After normalizing for β-actin and then for the mean of SELENBP1 in normal myometrium, data derived from densitometry of Western Blot experiments were used to represent relative levels of SELENBP1. Larger leiomyomas appeared to show a lower level of SELENBP1 than smaller ones, but no statistical difference in SELENBP1 levels was found among the four arbitrary size groups. The vertical bars in B represent means and standard deviations. Abbreviations: LM, leiomyoma; MM, normal myometrium; SELENBP1, selenium-binding protein1; n, number of leiomyomas.

**Figure 3:**
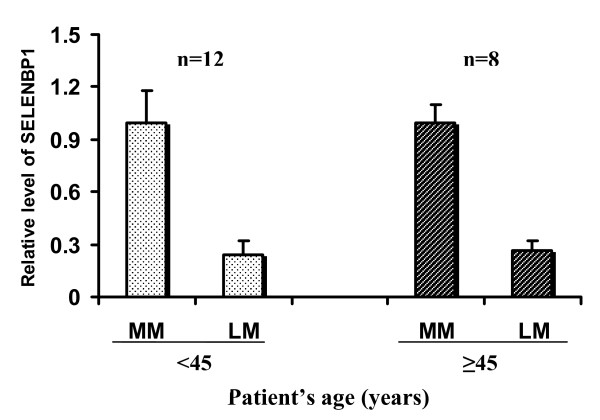
**Expression of SELENBP1 in normal myometrium and leiomyoma in patients younger than 45 years and older patients**. After normalizing for β-actin and then for the mean of SELENBP1 in normal myometrium, data derived from densitometry of Western Blot experiments were used to represent relative levels of SELENBP1. Although the level of SELENBP1 in leiomyoma was significantly lower than that in normal myometrium within either <45 years group (p = 0.0004883) or within ≥45 years group (p = 0.007812), no difference in SELENBP1 level was seen between the two age groups (p = 0.7576 in normal myometrium and p = 0.3749 in leiomyoma). The vertical bars represent means and standard deviations. Abbreviations: LM, leiomyoma; MM, normal myometrium; SELENBP1, selenium-binding protein1; n, number of cases.

**Figure 4:**
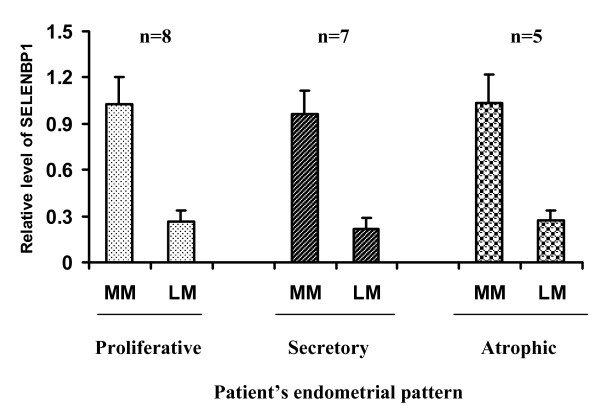
**Expression of SELENBP1 in patients with different endometrial patterns**. After normalizing for β-actin and then for the mean of SELENBP1 in normal myometrium, data derived from densitometry of Western Blot experiments were used to represent relative levels of SELENBP1. The level of SELENBP1 in leiomyoma was significantly lower than that in normal myometrium in patients with proliferative endometrium (p = 0.00782), secretory endometrium (p = 0.01562), or atrophic endometrium (p = 0.0447); however, no difference in SELENBP1 level was found among patients of different endometrial patterns (p = 0.43079 in normal myometrium and p = 0.91153 in leiomyoma). The vertical bars represent means and standard deviations. Abbreviations: LM, leiomyoma; MM, normal myometrium; SELENBP1, selenium-binding protein1; n, number of cases.

We performed Western Blot analysis on one sample of normal myometrium and one sample of each leiomyoma in any patient, with a total of 20 samples of normal myometrium and 73 samples of leiomyoma. Western Blot analysis using anti-human SELENBP1 recognized a single band at 56 kDa in all samples examined. The intensity of bands in leiomyoma was about 4-fold lower than that in normal myometrium (examples in two patients are shown in Figure 1A), and the difference was statistically significant (Figure 1B). Although there was a trend for a decreased expression of SELENBP1 with increasing tumor size (Figure 2), no statistical difference was seen among four arbitrary size groups: ≤2 cm, 2.1-5 cm, 5.1-8 cm, and ≥8 cm. The levels of SELENBP1 did not differ between patients younger than 45 years and older patients in either normal myometrium or leiomyoma (Figure 3). However, the level of SELENBP1 was significantly lower in leiomyoma than in normal myometrium either in patients younger than 45 years or in older patients. SELENBP1 expression did not differ among patients with proliferative, secretory, and atrophic endometrium either in normal myometrium or leiomyoma (Figure 4), but leiomyoma showed a significantly lower level of SELENBP1 than normal myometrium either in patients with proliferative endometrium, in patients with secretory endometrium, or in patients with atrophic endometrium.

**Figure 5:**
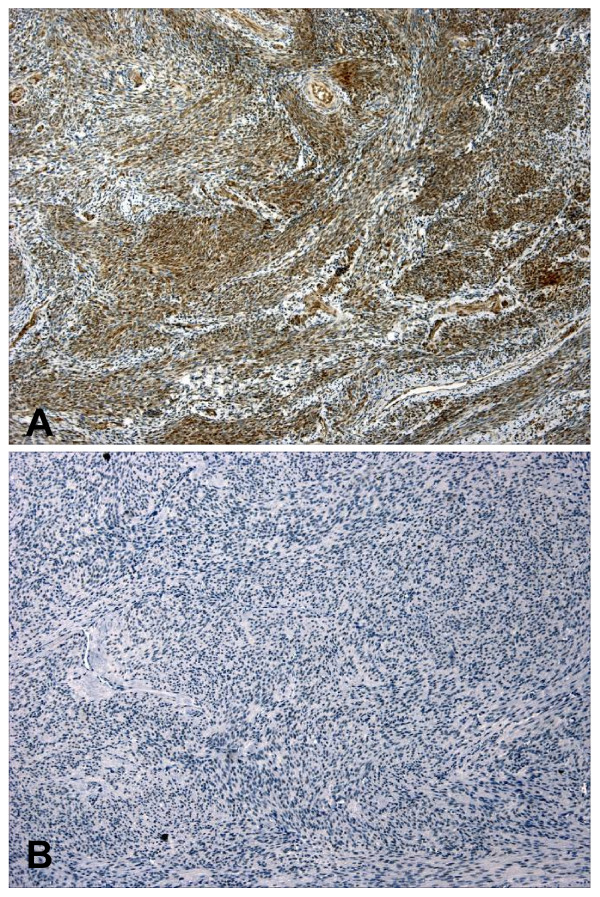
**Immunohistochemical studies of selenium-binding protein 1 expression**. Diffuse immunostaining was seen in normal myometrium (A), but uterine leiomyoma showed a near complete loss of immunostain (B).

Immunohistochemistry was performed on one section of normal myometrium and one or more sections of leiomyoma in each case, with a total number of 20 sections of normal myometrium and 42 sections of leiomyoma. Normal myometrium showed diffuse and strong staining (Figure 5); in a scale of 0-3, the staining scores ranged from 2 to 3 (mean = 2.3). The staining scores in leiomyoma were predominantly 0 and 1 with only occasional 2 (mean = 0.8). The difference in immunostaining scores between normal myometrium and leiomyoma was statistically significant (p = 0.00357); this result confirmed the finding by Western Blot analysis. No difference in staining was seen between patients younger than 45 years and older patients in either normal myometrium (p = 0.3285) or leiomyoma (p = 0.4596). The staining did not differ among patients with proliferative, secretory, and atrophic endometrium in either normal myometrium (p = 0.2806) or leiomyoma (p = 0.4736). Because size of leiomyoma was not specified for most sections used in immunohistochemistry, we were not able to correlate tumor size with immunostaining.

Among 42 available sections of leiomyomas, the vascular count varied from 24 to 96 per 5 low power fields with a mean of 38 per 5 low power fields. Statistic analysis did not reveal a significant correlation between the vascular counts and the SELENBP1 immunostaining scores (r = -0.0226, p = 0.4668). Proliferation index was similar among all leiomyomas and ranged from 0 to 6% (mean 1.2%). There was no significant correlation between the proliferation index and the SELENBP1 immunostaining score (r = -0.1562, p = 0.2668).

## Discussion

Our study showed a significant decrease of SELENBP1 in uterine leiomyoma than in normal myometrium. To our knowledge, this is the first study to examine SELENBP1 expression in normal myometrium and uterine leiomyoma. The presence of SELENBP1 in myometrium indicates a normal biologic function of SELENBP1 in this tissue. SELENBP1 has been implicated to play a role in toxification/detoxification processes [27], cell growth regulation [28], and intra-Golgi protein transport [29]. The decrease of SELENBP1 expression in uterine leiomyoma suggests that SELENBP1 is related to the development of this tumor. Although loss of SELENBP1 expression may be secondary to tumor development, the ability of SELENBP1 to inhibit cell proliferation and induce apoptosis in colon cancer [30] may suggest that the protein may also be involved in tumorigenesis of uterine leiomyoma. It is likely that additional genetic and molecular events contribute to tumorigenesis, but reduction of SELENBP1 expression may be a key step in the transition from normal myometrium to leiomyoma. Indeed, decreased expression of SELENBP1 in even the smallest leiomyoma (i.e., 1 cm) examined suggests that alteration in SELENBP1 expression may be an early event in the development of the tumor.

The mechanisms for tumorigenesis of SELENBP1 in uterine leiomyoma are not known. The development and growth of uterine leiomyoma has been attributed in part to estrogen stimulation; leiomyoma enlarges during pregnancy when estrogen level is high and in women taking tamoxifen or receiving estrogen-replacement therapy but shrinks in patients with low level of estrogen after treatment with gonadotropin-releasing hormone. In breast cancer cells, selenium has been shown to disrupt estrogen signaling pathway by decreasing the expression of estrogen receptors, decreasing the binding of estradiol to estrogen receptor, inhibiting the trans-activating activity of estrogen receptor, and reducing the binding of estrogen receptor to the estrogen responsive element site [31]. In myometrium showing normal expression of SELENBP1, selenium may be able to disrupt estrogen signaling pathway. When the expression of SELENBP1 is reduced, however, leiomyoma may develop because selenium without adequate SELENBP1 may be incapable of inhibiting the stimulatory actions of estrogens.

Sex hormones change in abundance through menstrual cycle. In women of reproductive age, estrogen dominates in the proliferative phase, and progesterone rises in the secretory phase. In postmenopausal women, estrogen and progesterone levels are low. Our study demonstrated similar levels of SELENBP1 among patients with proliferative, secretory, and atrophic endometrium in either normal myometrium or leiomyoma, indicating that SELENBP1 is not regulated by sex hormones. Also, our study did not find a difference in SELENBP1 level between patients younger than 45 years and older patients. In general, younger women are associated with a higher level of estrogen and progesterone than older women. Thus the same level of SELENBP1 regardless of age again suggests that SELENBP1 level is not under the influence of sex hormones.

A negative correlation between SELENBP1 expression and Ki67 positivity has been reported in lung adenocarcinomas [24], but our study did not reveal a significant relationship between SELENBP1 expression and proliferation index in uterine leiomyomas. Our result may be explained by a similarly low proliferation index among all uterine leiomyomas examined. While no previous report has addressed the relationship between vascular count and SELENBP1 expression in any tumor, we showed that vascular count was not correlated with SELENBP1 expression in uterine leiomyomas.

Medical treatment of uterine leiomyoma involves the use of gonadotropin-releasing hormone that inhibits steroidogenesis, induces chemical menopause, and therefore can reduce tumor volume with an improvement in clinical symptoms. However, the effects are short-lived, and leiomyoma tends to grow back rapidly after cessation of therapy. Lack of available effective medical therapy has made surgery the mainstay of treatment. The complications of surgery could be severe, particularly for young women who wish to preserve their fertility. Therefore, searching for novel target-based preventive and therapeutic agents has become imperative. Selenium has been implicated as an important chemopreventive and chemotherapeutic agent for several epithelial tumors, including cancers of the prostate and colon [32-34]. Our study showing a decreased expression of SELENBP1 in uterine leiomyoma not only indicates a role of SELENBP1 in tumorigenesis but also suggests the potential utility of selenium in prevention and treatment of uterine leiomyoma. Since the effects of selenium are mediated by SELENBP1, loss of SELENBP1 expression in leiomyoma may have a negative impact on the ability of selenium to control tumor cell growth. It has been reported, however, that treating ovarian tumor cells with a selenium compound increases SELENBP1 expression [35]. Thus the increased level of SELENBP1 after selenium treatment may facilitate the effect of selenium.

In summary, our study showed a decreased level of SELENBP1 in uterine leiomyoma compared to normal myometrium and suggested a role of SELENBP1 in tumorigenesis of leiomyoma. Our findings may provide a basis for future studies concerning the molecular mechanisms of SELENBP 1 in tumorigenesis as well as the potential use of selenium as a preventive and therapeutic agent in uterine leiomyoma.

## Competing interests

The authors declare that they have no competing interests.

## Authors' contributions

PZ carried out the Western Blot and immunohistochemistry studies, performed the statistical analysis, and participated in manuscript writing. CZ designed the study and wrote the manuscript. XW collected study samples and reviewed manuscript. FL, CJS, MRQ, and WDL participated in study design and reviewed manuscript. All authors read and approved the final manuscript.
